# Hepcidin and arterial stiffness in children with systemic lupus erythematosus and lupus nephritis: A cross-sectional study

**DOI:** 10.1371/journal.pone.0214248

**Published:** 2019-03-29

**Authors:** Meredith A. Atkinson, Sarah Joo, Sangeeta Sule

**Affiliations:** 1 Department of Pediatrics, Johns Hopkins University School of Medicine, Baltimore, Maryland, United States of America; 2 Kaiser San Francisco, San Francisco, California, United States of America; 3 Division of Rheumatology, Children’s National Medical Center, Washington, District of Columbia, United States of America; Lady Davis Institute for Medical Research, CANADA

## Abstract

**Background:**

Cardiovascular disease is common in patients with systemic lupus erythematosis (SLE) and lupus nephritis (LN). Up to 80% of children with SLE develop kidney disease, which is also associated with increased risk for cardiovascular disease and death compared to those without renal involvement. Hepcidin is an iron-regulatory protein which may contribute to atherosclerosis and is elevated in autoimmune disease. Pulse wave velocity (PWV) is a validated indicator of arterial stiffness, an early marker of cardiovascular risk, and is increased in children with SLE versus healthy controls. Our objective was to quantify hepcidin and PWV in children with SLE and investigate if those with biopsy-proven LN have higher hepcidin levels and higher PWV compared to those without kidney disease.

**Methods:**

Cross-sectional analysis with hepcidin was measured via ELISA assay in 16 children aged 10–21 years with SLE recruited from a single center. Subjects were classified as having LN if histologic evidence of the disease was documented on a clinical renal biopsy. Serum hepcidin was quantified using a validated competitive enzyme-linked immunoassay. Carotid-femoral PWV was measured using applanation tonometry. Wilcoxon rank sum testing was used to compare median levels of hepcidin, PWV, and other continuous variables by nephritis status.

**Results:**

The cohort (n = 16) was 93.8% female and 68.8% African-American with mean (SD) 16 (3.6) years. 37.5% (n = 6) had LN. Overall median (IQR) hepcidin was 34.4 (18.9, 91.9) ng/ml, and PWV 4.4 (4, 4.6) meters/second. Although significance was limited by small sample size, both hepcidin and PWV were higher in the subjects with LN. Median (IQR) hepcidin in subjects with LN was 71.5 (26.4, 116.4) ng/ml compared to 27.9 (18.7, 59.7) ng/ml in those with SLE(p = 0.19). Similarly, median (IQR) PWV in those with LN was 4.4 (4.4, 4.9) meters/second compared to 3 (3.75, 4.55) meters/second in those with without kidney involvement (p = 0.10).

**Conclusion:**

PWV and serum hepcidin were higher in subjects with LN compared to those with SLE alone, suggesting that elevated hepcidin levels may be associated with morbid CV changes in children with LN. This association, along with identification of additional predictors of arterial stiffness in patients with LN, warrants further investigation.

## Introduction

Children with systemic lupus erythematosus (SLE) are significantly more likely to develop lupus nephritis (LN) than SLE-affected adults, and 80% of pediatric SLE patients will have kidney involvement at some point in the course of their disease [1, 2]. The spectrum of kidney disease in SLE ranges from hematuria and proteinuria to diffuse proliferative glomerulonephritis leading to chronic kidney disease (CKD) and end stage renal disease (ESRD) [1,3]. Despite immunosuppressive therapy, only slightly more than half of patients with childhood-onset SLE who develop LN with proliferative glomerular lesions achieve a renal remission, leaving them at high risk for the development of ESRD [4–6]. The development of LN in children with SLE is also associated with significantly increased risk for long-term morbidity and mortality. Among children with ESRD, those with lupus nephritis as the underlying cause are at increased risk for hospitalization compared to children with ESRD from other causes [1,3], and also demonstrate twice the risk of death, with the most common causes being cardiovascular disease and cardiac arrest [1].

Both adults and children with SLE are at increased risk for atherosclerosis, which does not appear to be related to classical cardiovascular risk factors such as hypertension and hypertriglyceridemia, but rather appears to be mediated by inflammation related to underlying disease [7,8]. The inflammation-associated, iron-regulatory protein hepcidin has emerged as a key mediator of anemia in ESRD, and also likely contributes to the development of anemia in SLE. Evidence is now emerging that hepcidin may also contribute to the increased risk for cardiovascular mortality seen in children with ESRD secondary to SLE.

Hepcidin production in the liver is directly upregulated by pro-inflammatory cytokines including IL-6, which are increased in the setting of active SLE, and cleared from the circulation by glomerular filtration [9,10]. Hepcidin causes internalization and degradation of the primary cellular iron exporter, ferroportin, resulting in trapping of stored iron within macrophages and other iron storing cells [9,10]. Serum hepcidin levels are increased in both adults and children with CKD and ESRD, and are associated with erythropoiesis stimulating agent resistant anemia due to the inability of stored iron to be accessed for erythropoiesis [11]. Serum hepcidin levels are also increased in adults with autoimmune diseases including SLE and rheumatoid arthritis [12,13]. Atherosclerotic lesions typically contain macrophages laden with iron, which subsequently are critical determinants of lesion progression and stability [8]. Increased iron deposition in atherosclerotic lesions associated with increased hepcidin expression may contribute to the increased prevalence of atherosclerosis seen in patients with SLE [8]. Carotid-femoral pulse wave velocity (PWV) is a validated indicator of central arterial stiffness, a vascular wall phenotype which is an early marker of cardiovascular risk in children [14]. PWV can be measured non-invasively, is strongly correlated with cardiovascular events and mortality, and has been found to be elevated in children with SLE compared to healthy controls [7].

There have been no studies that we are aware of investigating serum levels of hepcidin and cardiovascular risk factors simultaneously in a pediatric SLE cohort. The goal of this investigation was to quantify serum hepcidin levels and PWV in a cohort of children with SLE followed at a single center, and to investigate whether children with biopsy-proven LN demonstrate higher serum hepcidin levels and increased PWV compared to those without kidney disease. Observation of higher hepcidin levels in children with SLE, and particular in children with lupus nephritis, may suggest not only that the use of hepcidin-antagonists could have a role in the treatment of the anemia of SLE in those with kidney disease, but also that such agents could interrupt the pathway for increased cardiovascular mortality in children with CKD and ESRD secondary to SLE.

## Materials and methods

Sixteen children and adolescents aged 10–21 years with SLE who were seen in the outpatient Pediatric Rheumatology or Nephrology clinics for routine follow-up visits at the Johns Hopkins Children’s Center between June 2014 and January 2015 were included in the analysis. Recruitment was via in-person approach of eligible subjects and parents/guardians during the visit. The study was approved by the Johns Hopkins Institutional Review Boards. All participants ≥ 18 years of age provided written informed consent; for children < 18 years, written informed consent was provided by a parent/legal guardian. Demographic data including age, sex, and race (African-American, Caucasian, Asian, or Other) data was obtained from subjects, parent/legal guardian, and/or the Johns Hopkins electronic medical record. Participants provided a serum sample which was frozen and stored at -80°C. Exclusion criteria included decreased estimated glomerular filtration rate (eGFR) of less than 90 ml/min/1.73m^2^, or ESRD requiring renal replacement therapy (dialysis or kidney transplant). Creatinine was measured locally in the Johns Hopkins Clinical Laboratory via the enzymatic method, and eGFR was calculated using the Schwartz equation [15].

Previously frozen serum samples were batch shipped overnight to Intrinsic LifeSciences (La Jolla, California) and hepcidin was quantified in all subjects using a validated competitive enzyme-linked immunosorbent assay (ELISA), with sensitivity of 5 ng/mL and inter-assay variability of 12% in a single laboratory [16]. Hemoglobin (Hgb) was measured locally in the Johns Hopkins Clinical Laboratory, and anemia was defined as hemoglobin value less than the 5^th^ percentile for age and sex [17]. Blood pressure was measured manually with subjects seated using an aneroid sphygmomanometer. Subjects were classified as taking anti-hypertensive medication if they were prescribed a daily angiotensin-converting enzyme inhibitor, calcium channel blocker, beta blocker, vasodilator, or alpha agonist medication for management of elevated blood pressures as indicated by the electronic medical record and confirmed by the subject and family. During the same study visit at which serum was collected, carotid-femoral PWV in meters/second (m/s) was measured non-invasively by applanation tonometry using the SphygmoCor system (AtCor Medical, Sydney, Australia) using methods that have previously been described [14]. Thirteen subjects had successful PWV measurements obtained; of the 3 subjects missing PWV data, 1 had LN and 2 did not. Subjects were classified as having LN if they had evidence of the disease on a kidney biopsy performed for clinical indications.

### Statistical considerations

Statistical significance was p < 0.05. All analyses were performed using Stata version 14.0 (Stata Corporation, College Station, TX). Distributions of continuous variables were assessed for normality, and summary statistics are presented as median (IQR) or proportions. Pearson chi-squared testing was used for comparisons of proportions between groups. Wilcoxon rank sum test was used to compare medians of hepcidin levels and PWV along with other continuous variables in children with SLE with and without lupus nephritis. Pearson’s correlation was used to assess the association between hepcidin and PWV. A power calculation for comparison of two means was conducted for the number of subjects enrolled in the study with alpha = 0.05 (two-sided).

### Ethics approval and consent to participate

This study received ethics approval from the Johns Hopkins Medicine Institutional Review Boards (IRB00032934). All subjects had a parent or guardian provide written informed consent for this study.

## Results

**Fig 1:**
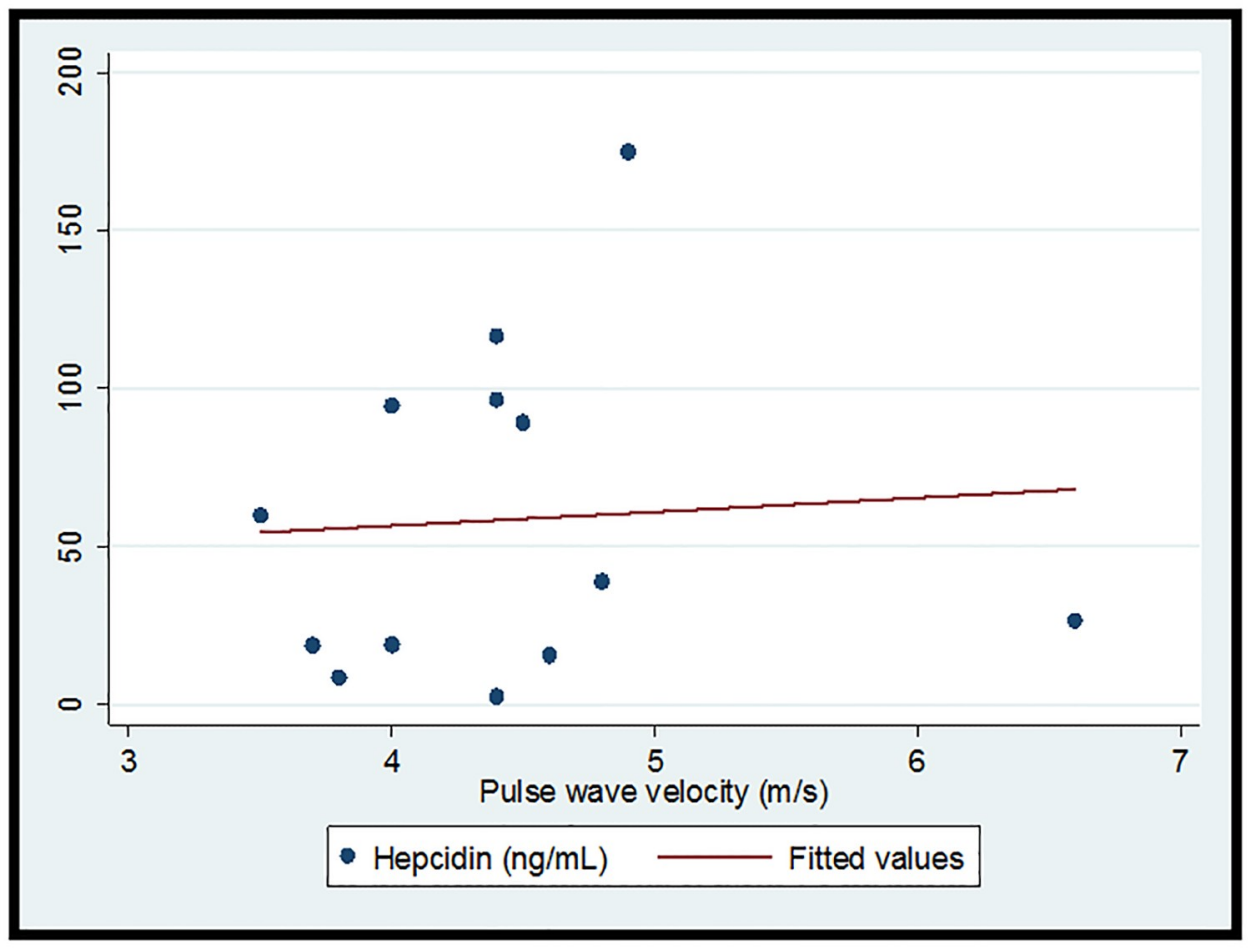
Correlation and linear prediction plot for hepcidin and pulse wave velocity in children with SLE. r = 0.06, p = 0.83.

**Fig 2:**
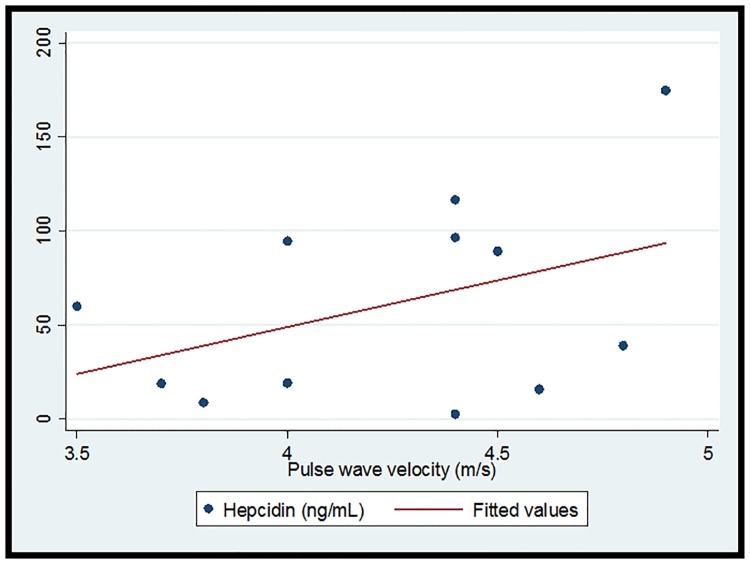
Correlation and linear prediction plot for hepcidin and pulse wave velocity in children with SLE, outlier value excluded. r = 0.41, p = 0.18.

**Fig 3:**
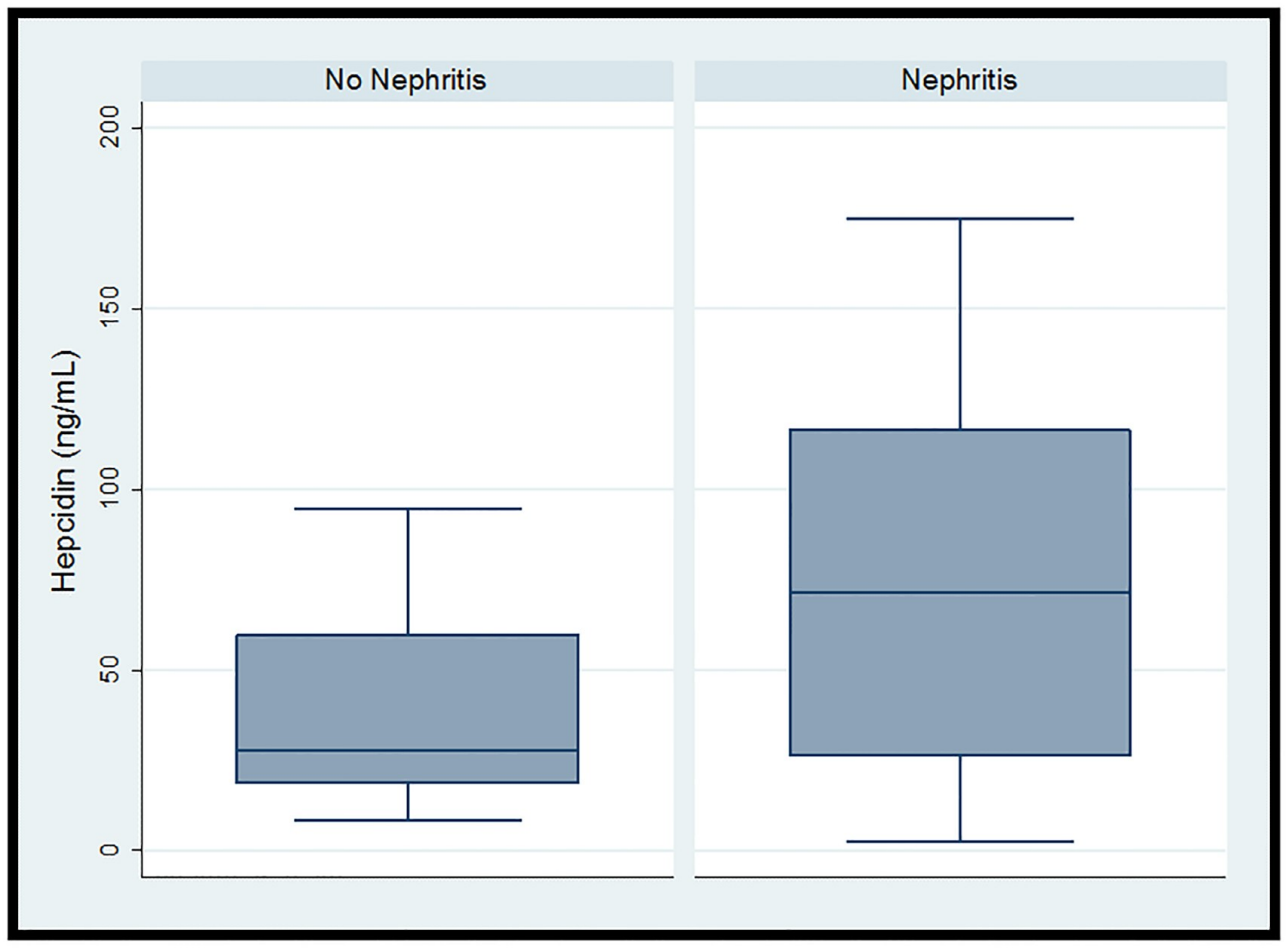
Boxplots of serum hepcidin and pulse wave velocity in children with systemic lupus erythematosus by nephritis status.

The cohort (n = 16) was 93.8% (n = 15) female and 68.8% (n = 11) African-American with median (IQR) age 16 (11.5, 18)) years. 37.5% (n = 6) had LN. Overall median (IQR) hepcidin was 34.4 (18.8, 91.8) ng/ml, and hemoglobin was 12.2 (11.6, 12,7) g/dl. Median (IQR) PWV in the 13 subjects with successful measurements obtained was 4.4 (4, 4.6) m/s. Overall there was no significant correlation between hepcidin and PWV (r = 0.06, p = 0.83) (Fig 1). One subject had a measured PWV value above the third quartile plus 1.5 the interquartile range. In a subsequent correlation analysis when this point of high influence was excluded, a trend toward a positive correlation emerged (r = 0.41, p = 0.18) (Fig 2). Table 1 demonstrates demographic and clinical data by LN status. Notably, all subjects with LN were African-American females. Although significance was limited by small sample size, the point estimates for both serum hepcidin and measured PWV were higher in the subjects with LN. A post-hoc power calculation demonstrated 27% power to detect the observed differences in hepcidin and 45% power to detect the observed differences in PWV, confirming that small sample size limited our ability to detect significant differences. Fig 3 depicts serum hepcidin and PWV by nephritis status.

**Table 1:** Demographic and clinical characteristics by nephritis status in 16 children and adolescents with systemic lupus erythematosus.

Median (IQR) or n (%)	No Nephritis (n = 10)	Nephritis (n = 6)	p-value
**Age (years)**	15.5 (11, 18)	17 (15, 19)	0.33
**Female**	9 (90%)	6 (100%)	0.42
**Race, n (%)**			0.23
***African-American***	5 (50%)	6 (100%)	
***Caucasian***	3 (30%)	0 (0%)	
***Asian***	1 (10%)	0 (0%)	
***Other***	1 (10%)	0 (0%)	
eGFR*, **ml/min/1.73m^2^**	139.5 (130, 193)	136.5 (102, 182)	0.33
**Hepcidin, ng/ml**	27.9 (18.7, 59.7)	71.5 (26.4, 116.4)	0.19
**Hemoglobin, g/dl**	12.2 (11.7, 12.6)	12 (11.2, 12.8)	0.91
Anemic**[18], **n (%)**	3 (30%)	3 (50%)	0.42
**On anti-hypertensive medication**	0 (0%)	5 (83.3%)	<0.001
**Systolic blood pressure, mmHg**	106 (104, 116)	122 (118, 128)	0.14
**Diastolic blood pressure, mmHg**	58 (58,68)	65 (60, 68)	0.31
**Pulse wave velocity, m/s**	3 (3.75, 4.55)	4.4 (4.4, 4.9)	0.10

*Estimated glomerular filtration rate;

**Defined as hemoglobin less than the 5^th^ %ile for age and sex

All subjects in the cohort had normal renal function with eGFR > 90 ml/min/1.73m^2^, and there was no significant difference in eGFR by nephritis status. Median hemoglobin did not differ by nephritis status, nor did the prevalence of anemia. More subjects with nephritis were prescribed antihypertensive medication compared to those without nephritis, 83.3% vs. 0%, p<0.001. However, differences in systolic and diastolic blood pressure between groups did not reach statistical significance.

## Discussion

The incidence of morbidity and mortality in SLE patients of all ages has a bimodal distribution, with early mortality seen in the context of active lupus or infectious complications in the setting of immunosuppression, and later onset mortality due to morbid cardiovascular changes [7,18,19]. Young adults with SLE in particular have significantly increased cardiovascular risk compared to age-matched counterparts in the general population. Among younger women aged 18–44 years, who are disproportionately affected by SLE, risk for hospitalization for myocardial infarction or stroke were more than twice that seen in healthy women [20]. In a study of pre-menopausal women with SLE there was a 50-fold higher incidence of myocardial infarction compared to healthy women [21]. Many of the cardiovascular risk factors specific to SLE likely have their origins in childhood, and the presence of kidney disease magnifies these risks; both adults and children with ESRD secondary to LN have increased for mortality compared to patients with ESRD from other causes [1]. We have shown that in a small cohort of children with SLE, those with LN demonstrated a trend toward higher serum hepcidin and PWV values compared to those without kidney involvement.

This pilot study aimed to explore the relation between hepcidin and PWV as a surrogate marker for cardiovascular risk (e.g. arterial stiffness) in systemic lupus erythematosus pediatric patients, particularly in those with lupus nephritis. Levels of the inflammation-associated, iron-regulatory protein hepcidin have been reported to be elevated in patients with autoimmune disease including SLE and rheumatoid arthritis, but this has not been explored in children with SLE [8,13,22]. There is limited data defining normal values for serum hepcidin in children, but a study in 86 healthy children reported a mean (SD) level 40.8 (13.9) ng/ml overall, with median (IQR) 36.4 (28.5, 45.7) in females and 43.6 (32, 52.7) in males [23]. In our analysis, hepcidin levels in those with SLE without kidney disease were comparable to those reported in healthy children, while the distribution of hepcidin levels in subjects with LN was higher.

Hepcidin is a key mediator of the anemia of inflammation and CKD, and is also elevated in both adults and children with CKD and ESRD [10,11,24]. In addition to being elevated in the setting of inflammation, hepcidin is cleared from the circulation by glomerular filtration and thus is also increased in the setting of decreased kidney function [10]. For this analysis we included only subjects with normal renal function by eGFR, and notably found a trend for higher hepcidin levels in those with LN despite no significant difference in estimated renal function between groups, suggesting that there are eGFR-independent factors associated with higher observed hepcidin levels in these patients. Hepcidin is emerging as a potential mediator, via the “trapping” of iron within macrophages, of the increased risk for cardiovascular disease noted in patients with SLE [8]. The presence of iron-laden macrophages in atherosclerotic plaques may be associated with increased oxidative stress, lesion progression and destabilization, and the decreased mobilization of iron stored in ferritin out of macrophages mediated by hepcidin may contribute to this process [8, 25–27]. As atherosclerotic lesions become progressively unstable, the lesions themselves may act as an inflammatory stimulus for upregulation of hepcidin production [8]. Animal studies have demonstrated that iron chelation or iron-deficient diet can reduce the development of atherosclerotic lesions [25,26].

Arterial stiffness is associated with cardiovascular events and mortality in adults [14]. Models incorporating PWV as a measure of central arterial stiffness have suggested that a 1 standard deviation increment in PWV is equivalent to 10 years of aging [28]. Reference values for PWV in otherwise healthy children and adolescents have been previously published by Reusz et al. [29], and none of the subjects in our study demonstrated PWV values greater than the 95^th^ percentile for age and sex as defined in healthy children. Despite this, we did observe a trend for higher PWV in children with LN compared to those with SLE without kidney involvement. Sozeri et al. have previously reported that children with SLE demonstrate higher mean PWV than age- and gender-matched healthy controls (6.56 vs. 5.29 m/s—both values higher than the mean and median pwv values observed in our cohort), although they were not able to demonstrate a significant difference in PWV between those with SLE alone and the 27.4% of subjects with LN [7]. We did observe that 100% of subjects with LN were on anti-hypertensive medications, and hypertension is a well-established risk factor for cardiovascular disease in patients with SLE with or without kidney involvement [20]. Higher relative PWV may be an early indicator of subclinical atherosclerosis in individual patients, which may progress and contribute to excess risk for cardiovascular disease over time. CKD alone is associated with increased risk for cardiovascular disease, and children on dialysis have been shown to have increased PWV compared to healthy children [20,30]. Our observation of a trend toward higher PWV in children with LN suggests that the systemic and renal-specific effects of SLE may work additively to increase risk for cardiovascular disease, even before significant decline in kidney function is apparent.

Our observational study does have limitations, chief among which is the small sample size of the cohort recruited from a single center, which likely limited our ability to demonstrate statistical significance. As SLE is a relatively rare disease in children, clinical studies in this population are chronically challenged by sample size, and multi-center studies are critical to explore associations of cardiovascular risk in this group. However, our findings do suggest that the association between LN, hepcidin and arterial stiffness are worthy of further investigation in this population. We also did not have serum ferritin values available in subjects, which could have supported the hypothesis that hepcidin-mediated macrophage iron overload is associated with arterial stiffness in this group. We lacked data on inflammatory markers including ferritin, IL-6, or C-reactive protein to correlated with degree of anemia or hepcidin levels. Our cohort was overwhelmingly female, and those with LN all African-American, but this reflects the epidemiology of SLE in the population at large.

## Conclusions

PWV and serum hepcidin were increased in subjects with LN compared to those with SLE alone. These findings suggest that elevated hepcidin levels may be associated with morbid CV changes in patients with LN. This association, along with identification of predictors of arterial stiffness in patients with LN, warrants further investigation. Clarification of the specific contributors to cardiovascular morbidity in vulnerable children with SLE and LN is a crucial step toward identifying feasible, targeted interventions to mitigate this risk. Given the relative rarity of SLE and LN in children, future studies should take advantage of multicenter collaborations and registries to increase sample size and study power.

## Supporting information

S1 Data Stata dataset including data used in the present analysis. (DTA)

## Abbreviations

CKD: chronic kidney disease
ESRD: end stage renal disease
LN: lupus nephritis
PWV: pulse wave velocity
SLE: systemic lupus erythematosus
